# Auditory sensitivity to spectral modulation phase reversal as a function of modulation depth

**DOI:** 10.1371/journal.pone.0195686

**Published:** 2018-04-05

**Authors:** Emily Buss, John Grose

**Affiliations:** Department of Otolaryngology/Head and Neck Surgery, University of North Carolina at Chapel Hill, Chapel Hill, North Carolina, United States of America; Tokai University, JAPAN

## Abstract

The present study evaluated auditory sensitivity to spectral modulation by determining the modulation depth required to detect modulation phase reversal. This approach may be preferable to spectral modulation detection with a spectrally flat standard, since listeners appear unable to perform the task based on the detection of temporal modulation. While phase reversal thresholds are often evaluated by holding modulation depth constant and adjusting modulation rate, holding rate constant and adjusting modulation depth supports rate-specific assessment of modulation processing. Stimuli were pink noise samples, filtered into seven octave-wide bands (0.125–8 kHz) and spectrally modulated in dB. Experiment 1 measured performance as a function of modulation depth to determine appropriate units for adaptive threshold estimation. Experiment 2 compared thresholds in dB for modulation detection with a flat standard and modulation phase reversal; results supported the idea that temporal cues were available at high rates for the former but not the latter. Experiment 3 evaluated spectral modulation phase reversal thresholds for modulation that was restricted to either one or two neighboring bands. Flanking bands of unmodulated noise had a larger detrimental effect on one-band than two-band targets. Thresholds for high-rate modulation improved with increasing carrier frequency up to 2 kHz, whereas low-rate modulation appeared more consistent across frequency, particularly in the two-band condition. Experiment 4 measured spectral weights for spectral modulation phase reversal detection and found higher weights for bands in the spectral center of the stimulus than for the lowest (0.125 kHz) or highest (8 kHz) band. Experiment 5 compared performance for highly practiced and relatively naïve listeners, and found weak evidence of a larger practice effect at high than low spectral modulation rates. These results provide preliminary data for a task that may provide a better estimate of sensitivity to spectral modulation than spectral modulation detection with a flat standard.

## Introduction

Spectral information is thought to provide cues for the perception of both vowels and consonants [1–5]. Formants and formant transitions are associated with low-rate spectral modulation, whereas the harmonic structure of voiced speech is associated with higher-rate modulations. Speech recognition is thought to rely predominantly on relatively low-rate spectral modulations, in the region of 0.25 to 2 ripples per octave [2, 6, 7]. Sensitivity to spectral modulation predicts speech recognition among listeners with varying degrees of hearing loss and among cochlear implant users [3, 6, 8, 9]. Detection of spectral modulation, or discrimination between different patterns of spectral modulation, likely reflects a combination of frequency selectivity and the ability to process the pattern of intensity across frequency [4, 5, 10]. Based on the association between speech perception and sensitivity to spectral modulation, evaluating sensitivity to spectral modulation has been proposed as a clinically useful approach for evaluating auditory performance. More recently, sensitivity to spectral modulation has been evaluated in combination with temporal modulation [11–13].

Sensitivity to spectral modulation is typically assessed using one of two approaches: 1) estimating the spectral modulation depth at which listeners can detect that spectral modulation has been applied to a noise carrier, or 2) estimating the highest modulation rate for which listeners can detect a spectral modulation phase reversal. In both cases, spectral modulation is typically imposed via multiplication in the log frequency domain with a raised sinusoid, with spectral modulation rate defined in ripples per octave (rpo). In the spectral modulation detection paradigm, the standard stimulus is spectrally flat, and the target is spectrally modulated. Spectral modulation depth is adjusted to estimate the threshold for a particular modulation rate. In this paradigm, sensitivity to spectral modulation is characterized as the *spectral modulation detection threshold in dB*. In the spectral modulation phase reversal paradigm, both the standard and the target are spectrally modulated, but the modulation phase differs by 180°. Spectral modulation rate is adjusted to determine the highest rate at which a listener can discriminate modulation phase. In this paradigm, sensitivity to spectral modulation is characterized as the *spectral modulation phase reversal threshold in rpo*.

Anderson et al. [14] compared thresholds in these two paradigms–spectral modulation detection thresholds in dB and spectral modulation phase reversal thresholds in rpo–for three normal-hearing young adults. Spectral modulation detection thresholds were approximately 2.5 dB at 3 rpo, with poorer performance above and below that rate; notably, all three listeners were able to detect modulation at depths of less than 20 dB at 60 rpo, which was the highest rate tested. In contrast, spectral modulation phase reversal thresholds were approximately 8 rpo. In other words, the modulation detection paradigm indicated sensitivity at a higher rate of spectral modulation than the phase reversal paradigm. One possible explanation for this discrepancy in results across paradigms is that listeners were using a temporal cue–based on temporal beating between spectral peaks falling within an auditory filter–to detect high-rate spectral modulation. For example, spectral modulation of 16 rpo is associated with beats between neighboring spectral peaks ranging from approximately 15 Hz at the low-frequency edge of the stimulus (360 Hz) to 243 Hz at the high-frequency edge (5600 Hz). These rates are within the passband of sensitivity to envelope modulation on a noise carrier [15]. The value of temporal cues in the spectral modulation detection paradigm is complicated somewhat by the fact that spectral peak spacing is not uniform in Hz across frequency. As a consequence, envelope fluctuation is complex for filters passing more than two spectral peaks and varies in rate across auditory filters. The presence of beats would not provide a cue in the spectral modulation phase reversal task, as modulation is present in both intervals. Changes in modulation rate and the spectral distribution of temporal modulation cues differ as a function of spectral modulation phase, and theoretically listeners could use those differences to identify the target. However, behavioral data suggest that human listeners are not able to use these cues [14]. That result is consistent with data on modulation rate discrimination for a noise carrier. For the example of 16 rpo, spectral modulation phase reversal results in a 2% change in envelope modulation rate in neighboring spectral regions. While some data indicate that a 2% change in envelope modulation rate may be discriminable when applied coherently to a broadband masker, most data indicate thresholds >2% [16]; variability in envelope modulation depth and rate across frequency in the spectral modulation phase reversal task would further reduce the value of temporal cues.

There are advantages and disadvantages to using spectral modulation detection vs spectral modulation phase reversal for evaluating sensitivity to spectral modulation. One advantage of characterizing sensitivity by measuring spectral modulation detection thresholds in dB is that sensitivity can be evaluated at specific modulation rates. This could be beneficial in evaluating sensitivity to the low-rate modulation cues that support phoneme recognition. Spectral modulation phase reversal thresholds in rpo would indicate whether 100% modulation at these rates supported discrimination, but not the fidelity with which those cues were represented. One potential disadvantage of the spectral modulation detection paradigm is uncertainty regarding the contribution of temporal cues to performance [14].

The present report evaluates a hybrid approach for measuring sensitivity to spectral modulation. The listener’s task was to detect spectral modulation phase reversal, but the modulation depth (not the rate) was adjusted to determine threshold. This paradigm characterizes sensitivity to spectral modulation as *spectral modulation phase reversal threshold in dB*. While relatively uncommon, there is precedent for this approach in the literature [17]. The potential advantage of this method over the more commonly used paradigms, described above, is that it allows assessment of sensitivity at a particular rate of spectral modulation, without introducing salient temporal cues. Experiments were conducted to evaluate this potential advantage and provide preliminary data for this task across a range of conditions.

Experiment 1 measured performance as a function of modulation depth to determine appropriate units for adaptive threshold estimation. Experiment 2 compared thresholds in dB for two tasks: detection of spectral modulation, using a flat standard, and detection of modulation phase reversal. The goal was to replicate and extend the previous results of Anderson et al. [14]. Experiment 3 evaluated spectral modulation phase reversal thresholds for modulation that was restricted to either one or two neighboring octave-wide bands of noise. These stimulus conditions are relevant to speech recognition, where modulation is typically restricted in frequency. Experiment 4 measured spectral weights for spectral modulation phase reversal detection. Very little is known about normal-hearing listeners’ use of spectral modulation cues that are distributed across frequency, and this was deemed to be a fundamental feature of spectral modulation processing. Experiment 5 compared performance for highly practiced and relatively naïve listeners. Interest in this comparison is based on the observation that large practice effects could be problematic if this task were adapted for use as a clinical assessment tool.

## General methods

### Listeners

Listeners were adults with normal hearing, defined as pure-tone thresholds of 20 dB HL or less at octave frequencies 0.25–8 kHz [18]. No listener reported a history of hearing problems. Five of the listeners were highly practiced, with between 30 and 2000 hrs of prior psychoacoustic listening experience (25–63 yrs, mean of 41 yrs). The remainder were relatively inexperienced, many with no prior psychoacoustic listening experience (19–26 yrs, mean of 21 yrs). Experienced listeners participated in all five experiments, whereas naïve listeners provided data only for the final experiment. All listeners provided written consent to participate, and all procedures were approved by the Institutional Review Board of the University of North Carolina at Chapel Hill.

### Stimuli

Stimuli were composed of bands of pink noise, and each band either was or was not spectrally modulated. Spectral modulation, when present, was imposed via multiplication in the frequency domain with a raised sine wave, with both frequency and magnitude represented in log units (octaves and dB SPL, respectively). Across intervals within a trial, spectral modulation of the target was 180° out-of-phase with respect to spectral modulation of the standard. A new random starting phase for standard modulation was selected for each trial, and a new sample of pink noise was generated prior to each interval. Spectral modulation depth was defined as the peak-to-trough differences in dB.

**Fig 1:**
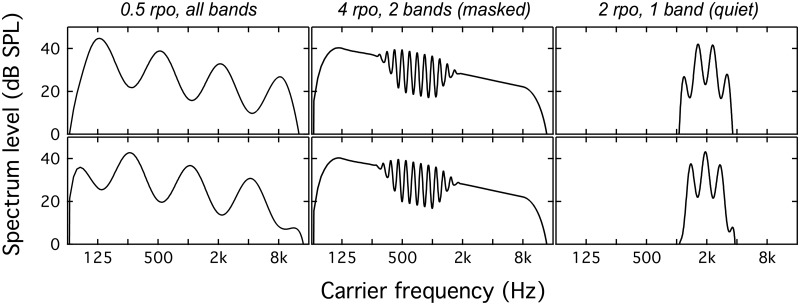
Examples of idealized magnitude spectra for stimuli in three conditions. In all cases the spectral modulation depth is 20 dB. The top and bottom rows show standard and target stimuli, respectively. The leftmost column shows 0.5 rpo for a condition in which all 8 bands are target bands. The middle column shows 4 rpo for a condition in which target bands are centered on 0.5 and 1 kHz, and the remaining bands are unmodulated noise (masked condition). The rightmost column shows 2 rpo for a condition in which the target is a single band centered on 2 kHz, and the remaining bands are scaled to zero (quiet condition). The +/- 6-dB interval rove is not depicted in this figure.

Stimuli were filtered into seven bands, with center frequencies 0.125 to 8 kHz, via multiplication with 2-oct-wide Hann windows. Windows were spaced at 1-oct intervals, and neighboring windows overlapped at the 6-dB-down points. The input to each of the eight filters could be pink noise, spectrally modulated pink noise, or an array of zeros (silence). Fig 1 illustrates idealized magnitude spectra for example stimuli in a selection of conditions; the top and bottom rows of panels represent pairs of standard and target stimuli. All stimuli were 168 ms in duration, including 20-ms raised-cosine ramps. While some studies of spectral modulation perception have used longer-duration stimuli, on the order of 400 ms [14, 19], there is precedent in the literature for using stimulus durations as short as 30–100 ms [20, 21]. Briefer stimuli may be preferable if they provide the listener with less robust temporal cues to spectral shape. Stimuli were presented at a median level of 70 dB SPL, with a trial-by-trial level rove of +/- 6 dB, based on a random draw from a uniform distribution.

### Procedure

Data were collected in a three-alternative forced-choice procedure, with a 400-ms inter-stimulus interval. Listeners indicated their responses by pressing one of three buttons on a handheld response box, and correct-answer feedback was provided visually. At the beginning of each data collection track three practice trials were provided, with 60-dB spectral modulation depth. Responses on these trials were not scored.

Performance was evaluated in two ways: by fixing the depth of spectral modulation and measuring percent correct (Exp 1 and 4) or by adaptively varying the modulation depth to estimate threshold (Exp 2, 3, and 5). Thresholds were measured using a 3-down, 1-up tracking rule that estimates 79% correct [22]. Initial adjustments in spectral modulation depth in dB were a factor of 2; that stepsize was reduced to 2^0.5^ after the second track reversal. Each track continued until eight reversals were obtained, and threshold was the geometric mean of the last six track reversals. The signal strength was capped at a depth of 60 dB. Any track that hit this limit six times was terminated early, and the threshold was reported as 60 dB. Modulation depth was adjusted in equal steps on units of log(dB); the rationale for using these unconventional units is discussed in more detail in the context of Experiment 1. Given the small number of listeners in some experiments, the results presented here should be viewed as preliminary.

## Experiment 1: Sensitivity to spectral modulation phase reversal as a function of modulation depth

The first experiment characterized the shape of the psychometric function by measuring listeners’ sensitivity to spectral modulation phase reversal as a function of spectral modulation depth. Three listeners provided data for each of five spectral modulation rates (0.25, 0.5, 1, 2 & 4 rpo). In preparation for this experiment, listeners completed four adaptive tracks at each spectral modulation rate, and mean thresholds were computed. The present experiment evaluated percent correct at each of five modulation depths, from 0.25 to 1.41 times the listener’s adaptive threshold. Six blocks of 60 trials each were collected for each spectral modulation rate. Within each block, trials cycled sequentially through the five depths relative to the listener’s threshold, from x1.41 (easiest) to x0.25 (hardest). The order of conditions was not randomized due to concerns that multiple very difficult trials in a row could result in the listener forgetting the target cue.

**Fig 2:**
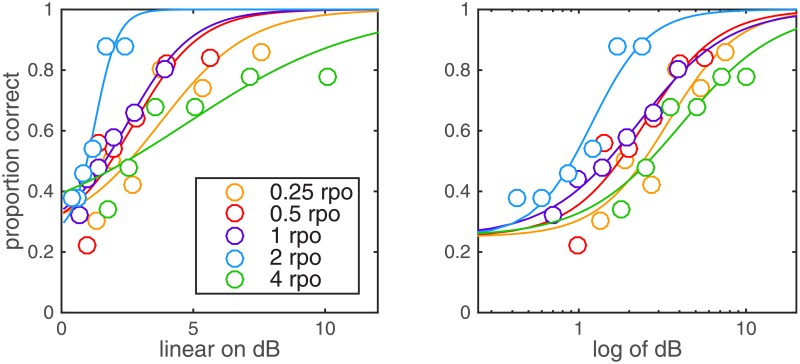
Psychometric functions by rate. Example fits are shown for one listener. Functions are plotted on dB modulation depth in the left panel and log(dB) in the right panel. Color reflects spectral modulation rate.

Data for each condition and each listener were fitted using a cumulative normal distribution, scaled by 2/3 and shifted up by 1/3 to capture effects of guessing in a three-alternative forced choice. Functions were fitted twice for each spectral modulation rate and listener, once in dB modulation depth and a second time in the log transform of that value. Example data for one listener are shown in Fig 2. For fits on log(dB), r^2^ = 0.52–0.99 (median r^2^ = 0.92). For fits on dB, r^2^ = 0.47–0.99 (median r^2^ = 0.89). These differences were not significant (F_1,2_ = 3.32, p = 0.210). For all five spectral modulation rates, the median psychometric function slope was broadly comparable on log(dB): performance rose from 40 to 85% correct with a factor of 4.1 (3.7–7.3) increase in modulation depth. Adaptive threshold estimates obtained using a 3-down, 1-up rule are affected by the magnitude of the stepsize relative to the psychometric function slope [15]. Adjusting modulation depth in log(dB) units supports the use of uniform stepsizes across conditions without introducing rate-dependent bias. Another reason to prefer units of log(dB) over units of dB is to avoid ceiling effects in the adaptive track. Results of this experiment support the use of factorial steps in the adaptive threshold procedures in subsequent experiments.

## Experiment 2: Thresholds in dB for modulation detection vs. phase reversal

**Fig 3:**
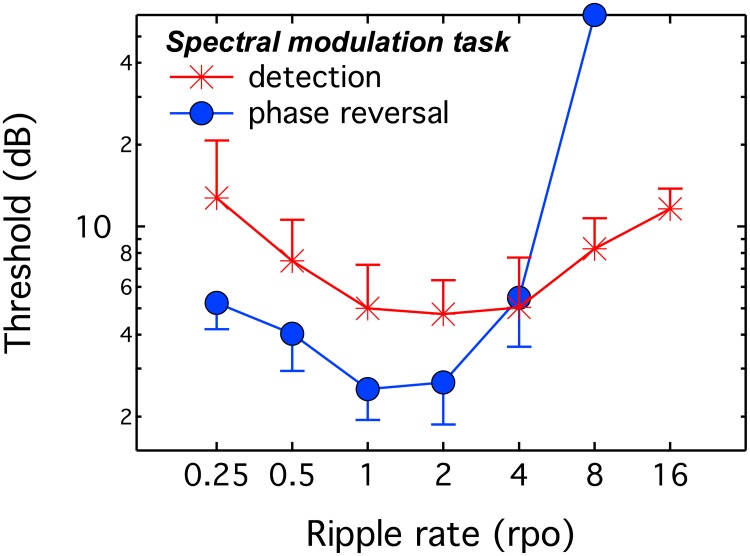
Thresholds as a function of spectral modulation rate for two tasks. Thresholds in dB are plotted for detecting spectral modulation and spectral modulation phase reversal. Error bars show one standard deviation, computed in log units.

Five highly practiced listeners provided data in two tasks: spectral modulation detection and spectral modulation phase reversal for rates of 0.25, 0.5, 1, 2, 4, 8, and 16 rpo, with threshold quantified in dB in both cases. Each listener provided three to four threshold estimates, as time allowed. As illustrated in Fig 3, performance in both tasks improved with increasing modulation rate up to 1–2 rpo. This result has been attributed to difficulties comparing spectral features that are distributed widely in frequency [21]. Above 2–4 rpo, thresholds worsened with increasing modulation rate, a finding attributed to detrimental effects of limited frequency resolution [19]. For modulation rates below 4 rpo, thresholds were lower for the phase reversal than the modulation detection task. This is consistent with the observation that differences in the magnitude spectra of target and standard stimuli are twice as large for the modulation phase reversal task than the modulation detection task. Thresholds for spectral modulation phase reversal were at the ceiling value of 60 dB by 8 rpo. In contrast, thresholds remained below 10 dB up to 16 rpo for the spectral modulation detection task. These results broadly replicate those reported by Anderson et al. [14].

The discrepancy between thresholds obtained for spectral modulation detection and spectral modulation phase reversal is consistent with the idea that temporal cues can be used to detect the introduction of high-rate spectral modulation, but that those cues are not beneficial in the phase reversal task. If temporal cues are available for a subset of the modulation detection conditions, one question of interest is which conditions reflect sensitivity to spectral modulation, and which reflect the use of temporal cues. If listeners were relying solely on changes in stimulus level across intervals at one or more fixed points in frequency, then threshold should be a factor of two poorer in the modulation detection task than the phase reversal task; this prediction is based on the observation that the change in local magnitude is twice as large in the phase reversal task as the modulation detection task. Thresholds in the modulation detection task differed from those in the phase reversal task by a factor of 2.4 (0.25 rpo), 1.9 (0.5 rpo), 2.0 (1 rpo), 1.8 (2 rpo), 0.9 (4 rpo), and 0.1 (8 rpo). Paired t-tests support the observation that effects of task are restricted to 4 rpo (p = 0.033) and 8 rpo (p < 0.001) after adjusting for the factor of two difference in stimulus differences across intervals. When looking for evidence of this result in Fig 3, recall that the x2 correction was not applied to those data points. While these values do not rule out the availability of temporal cues below 4 rpo, they suggest that temporal cues probably do not reliably improve spectral modulation detection below 4 rpo in normal-hearing listeners.

## Experiment 3: Carrier-frequency-specific spectral modulation phase reversal thresholds in dB

Whereas sensitivity to spectral modulation is often measured for spectral modulation across a wide range of frequency, the spectral cues required to identify speech are restricted in frequency (e.g., the region associated with formant frequencies). This is consistent with the observation that spectral shape discrimination is a better prediction of speech discrimination when the stimuli are restricted to the frequency region of the speech cue [23]. Processing frequency-specific spectral modulation cues may also be affected by the presence of off-frequency maskers. This experiment evaluated effects of carrier frequency and the presence of unmodulated off-frequency masking noise on spectral modulation phase reversal thresholds in dB.

Published findings on the effect of carrier frequency on sensitivity to spectral modulation are mixed. Eddins and Bero [19] report that spectral modulation detection thresholds in dB do not depend on carrier spectral region or bandwidth. In contrast, Supin et al. [17] found that spectral modulation phase reversal thresholds in dB do depend on the carrier frequency. That study evaluated listeners’ ability to detect a change in spectral modulation phase for a 3/4-octave-wide noise band. For 2.8 rpo, thresholds were similar for bands with center frequencies of 500 to 8000 Hz, with a mean of 3.3 dB. However, thresholds for higher-rate spectral modulation depended on the center frequency of the band. For example, 4.2-rpo spectral modulation phase reversal thresholds fell from 8.8 dB at 500 Hz to 4.8 dB at 4 kHz. Frequency effects for high-rate spectral modulation were argued to reflect variability of frequency resolution across frequency regions.

Supin and his colleagues have published several studies evaluating the effect of unmodulated noise on listeners’ sensitivity to spectral modulation imposed on a 1-oct band of noise. Quantifying sensitivity as the highest modulation rate supporting modulation phase reversal, they showed more masking for an unmodulated band below the target than above the target [24]. Under some conditions a masker below the target was more effective than an on-frequency masker [25]. Interestingly, increasing the bandwidth of the low-frequency masker did not introduce more masking–if anything, it decreased masking [24]. Masking in these studies was interpreted as reflecting a range of factors, including combination tones, upward spread of excitation, and/or lateral suppression [24, 26].

**Fig 4:**
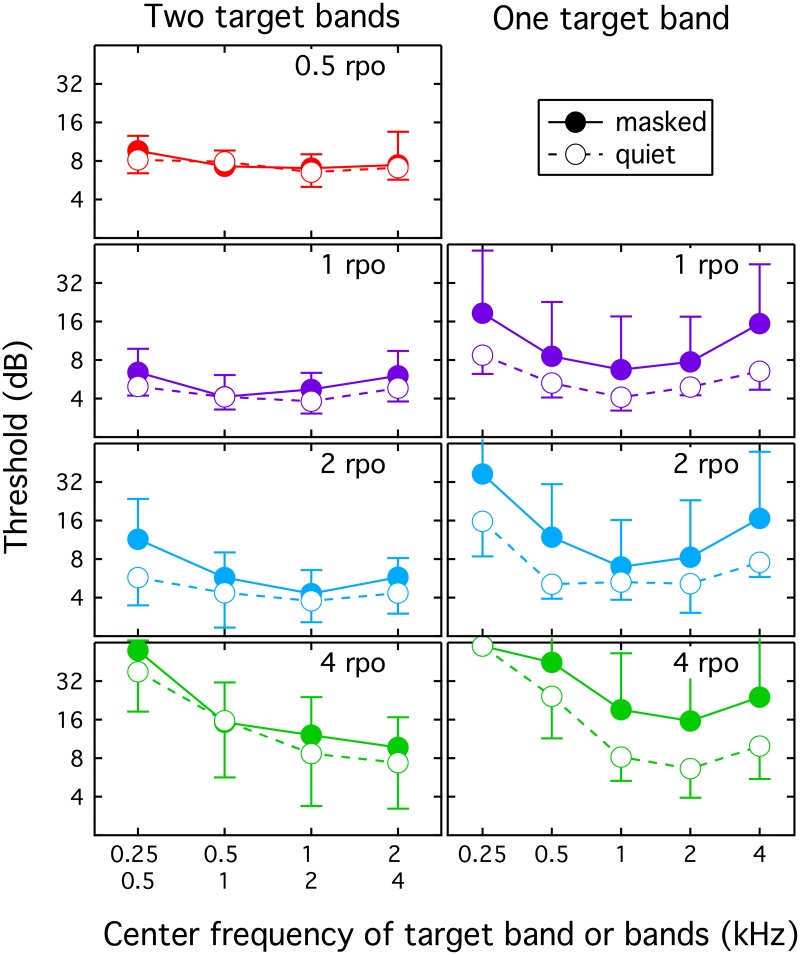
Thresholds for one- and two-band targets as a function of band center frequency. Thresholds are plotted as a function of band center frequency for targets spanning two octave-wide bands (left) or one octave-wide band (right). Filled symbols indicate thresholds obtained in the presence of unmodulated noise in the non-target bands (masked condition), and open symbols indicate that target bands were presented without flanking maskers (quiet condition). Each panel shows results for a different modulation rate, as indicated at the top of the panel. Error bars show one standard deviation, computed in log units.

In the present study, thresholds were collected for one- and two-band targets in different frequency regions, presented either in quiet or in the context of off-frequency bands of unmodulated noise. The target was never present in the lowest band (125 Hz) or the highest band (8000 Hz). A modulation rate of 0.5 rpo was not evaluated in the one-band condition because a 1-oct band would provide the listener with just one-half of a period of spectral modulation. The hypotheses were that detection thresholds for high-rate modulation would be better at high than low frequencies, as observed by Supin et al. [17], and that masking noise would have a more modest effect for the two-band than the one-band target, due to the more modest energetic masking in the spectral center of the two-band target. Three listeners provided data in all conditions, with three to four threshold estimates per condition. Mean thresholds are plotted as a function of carrier frequency in Fig 4.

Thresholds for the two-band signal were relatively unaffected by the presence of unmodulated neighboring bands, but there was a clear effect of carrier frequency at high spectral modulation rates. The relatively modest effects of presenting flanking bands that are not modulated can be seen in Fig 4 as a close correspondence between thresholds shown with filled and unfilled circles. Across conditions, flanking maskers elevated thresholds by a factor of 1.2 on average, with values ranging from 0.9 (0.5 rpo at 0.5 & 1 kHz) to 2.0 (2 rpo at 0.25 & 0.5 kHz). For both masked and quiet listening conditions, there was a trend for better high-rate modulation thresholds at higher carrier frequencies. This was most pronounced at 4 rpo, where thresholds fell from approximately 40 dB (0.25 & 0.5 kHz) to approximately 8 dB (2 & 4 kHz).

Thresholds for the one-band target were more clearly affected by the masker, although there were marked individual differences in susceptibility to masking. This can be seen in the right panels of Fig 4 in the separation between filled and unfilled circles, and the large error bars on the filled circles. Across conditions, flanking maskers elevated thresholds by a factor of 1.8 on average, with values ranging from 1.0 (4.0 rpo at 0.25 kHz) to 2.4 (4 rpo at 4 kHz). The modest effect of masking for 4 rpo at 0.25 kHz is due to the fact that performance with and without flanking maskers was at ceiling for all listeners. Similar to the two-band data for 2 and 4 rpo, there was a trend for thresholds to improve as the carrier was increased from 0.25 to 1 kHz. In contrast to the two-band data, however, there was also a trend for thresholds to rise again between 2 and 4 kHz. Based on these results, it is possible that the relatively low thresholds obtained in the two-band data for 2 & 4 kHz were based predominantly on cues in the 2-kHz band.

Results of this experiment contrast with those of Eddins and Bero [19], who found little evidence of a carrier frequency effect for spectral modulation detection. The present results are consistent with the frequency effects observed by Supin et al. [17], who reported poorer spectral modulation phase reversal thresholds for high-rate modulation at low than high carrier frequencies. The trend for best performance in the middle of the spectrum (1–2 kHz) in the one-band data is broadly consistent with the finding that sensitivity to increments in an otherwise flat spectral profile is greatest around 1 kHz [20].

## Experiment 4: Spectral weights for detecting spectral modulation phase reversal

The results of Experiment 3 are broadly consistent with better sensitivity to spectral modulation at 2 kHz than below that carrier frequency, particularly for high modulation rates. While the spectral region associated with the target was fixed across trials in the previous experiment, it is likely to be uncertain under natural listening conditions (e.g., when listening to speech in a dynamic masker). The present experiment therefore evaluated weights for spectral modulation phase reversal for conditions in which the spectral region associated with the best cues (largest modulation depth) varied randomly across trials.

**Fig 5:**
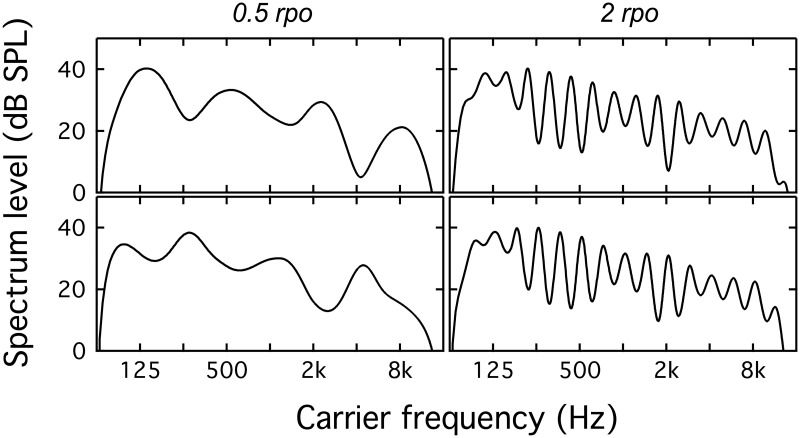
Examples of idealized magnitude spectra for 0.5 and 2 rpo. In both cases the nominal spectral modulation depth is 10 dB. The top and bottom rows show standard and target stimuli, respectively. The +/- 6-dB interval rove is not depicted in this figure.

Four highly practiced listeners provided data at a fixed nominal modulation depth for each spectral modulation rate (0.25, 0.5, 1, 2 & 4 rpo). The nominal depth used for each listener in each condition was selected to produce approximately 65% correct, based on previous adaptive threshold data and fixed-level pilot data, and this nominal depth was consistent across bands. In addition to the +/- 6-dB interval level rove used in all conditions in this series of experiments, the present experiment also incorporated band-specific jitter in the modulation depth associated with each trial. In this context, jitter refers to trial-by-trial variability in modulation depth. Prior to each trial, a random factor between 0.42 and 2.40 was identified for each of the seven spectral bands; a draw from a uniform distribution was used to select one of five values (0.42, 0.65, 1.00, 1.55, & 2.40), with equal probability. Those factors were applied to the spectral modulation depth associated with each band in each of the three listening intervals. Fig 5 illustrates idealized magnitude spectra for example stimuli, excluding the +/- 6-dB level rove. The nominal modulation depth for these examples is 10 dB. The top and bottom rows of panels represent pairs of standard and target stimuli, respectively, and the left and right columns show stimuli with 0.5 and 2 rpo. Listeners completed 10 blocks of 50 trials each. Spectral weights for spectral modulation phase reversal were estimated by evaluating the correlation between depth jitter in each band and correctness of the listener’s response. The hypothesis was that weights would generally resemble the differential sensitivity to spectral modulation for bands presented in quiet, with greater weight associated with regions of greater sensitivity.

**Fig 6:**
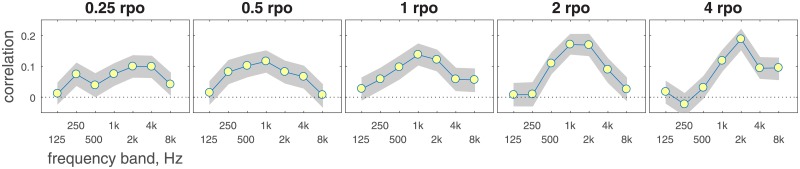
Correlation between spectral modulation depth and correct detection of modulation phase reversal. Correlations are plotted as a function of carrier band frequency, with results shown separately for each of the five spectral modulation rates. Symbols and lines indicate the correlation values based on 500 trials from each of four listeners. Shaded regions indicate the 95% confidence intervals around those estimates.

The pattern of weights was consistent across listeners, so only mean data are shown in Fig 6. Lines and symbols indicate the correlations in group data as a function of band frequency, and shading indicates the 95% confidence interval around each value, estimated using a bootstrapping procedure (n = 1000). For all five spectral modulation rates, bands at the low- and high-frequency edges of the stimulus (0.125 and 8 kHz) tended to receive less weight than bands intermediate to those edge frequencies. This is consistent with the observation that spectral modulation detection appears to be dominated by cues in the region associated with the best audiometric thresholds [6]. The pattern of weights was otherwise relatively flat for 0.25 rpo, consistent with the idea that listeners were using cues distributed across octave-wide bands in these conditions; this makes sense since at least four bands would be required to represent a full cycle of modulation at this low rate, and modulation jitter was imposed on 1-oct bands. There was increasing evidence of greater weight applied to the middle of the spectrum as the modulation rate increased. For 4 rpo there was a clear peak in weights at 2 kHz, and little or no weight applied to bands 0.125–0.5 kHz. In contrast, for the 0.5 rpo condition the 0.5 kHz band was among the bands receiving the highest weights. This is broadly consistent with data collected in quiet in Experiment 3, where performance was relatively consistent across frequency for low modulation rates but improved with increasing frequency up to 2 kHz for higher rates. The finding that weights varied across frequency contrasts with the findings of Eddins and Bero [19], who reported that thresholds for detecting spectral modulation do not depend on carrier spectral region bandwidth.

## Experiment 5: Ripple phase discrimination in naïve listeners

A clinical test of spectral modulation would need to be relatively consistent across normal-hearing listeners and immune to practice effects. While Eddins and Bero [19] found little evidence of practice effects, Sabin et al. [27] argued that practice may differ for different rates of spectral modulation. Listeners in that study underwent seven days of training on spectral modulation detection. Training produced the largest threshold improvement for 0.5 rpo, less at 1 rpo, and none at 2 rpo. In the final experiment, spectral modulation phase reversal thresholds in dB were compared for listeners with extensive listening experience (n = 5, Exp 2) and those with little or no prior listening experience (n = 15). Three to four threshold estimates were obtained at each of six spectral modulation rates, completed in a different random order for each listener.

**Fig 7:**
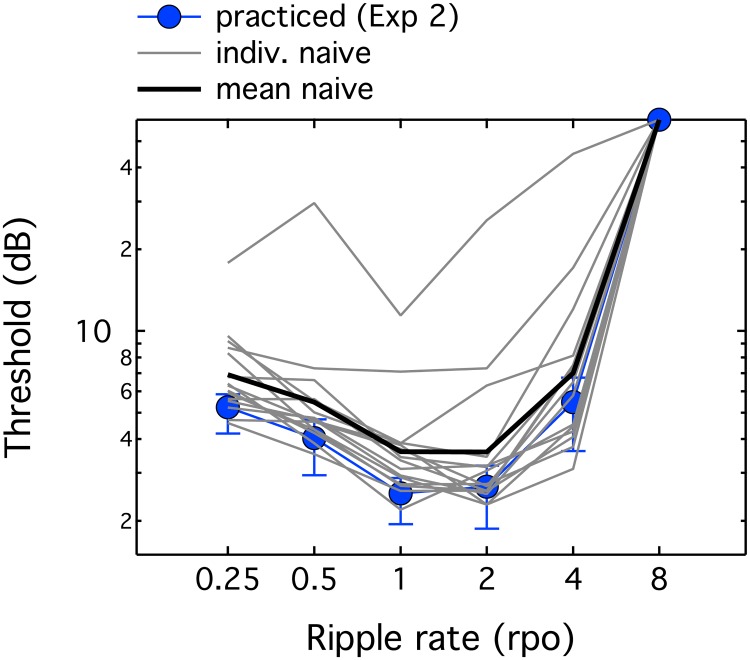
Thresholds as a function of spectral modulation rate. Thresholds for spectral modulation phase reversal in dB are plotted as a function of spectral modulation rate. Thin grey lines show thresholds for individual naïve listeners, and the thick black line shows the mean across naïve listeners. Circles show results from five highly practiced listeners (also shown in Fig 3).

Results are shown in Fig 7, with light grey lines showing thresholds for individual naïve listeners, and the solid black line showing the mean across naïve listeners. Blue circles show the mean results from practiced listeners. There was substantial variability across naïve listeners, with thresholds varying by x3.9 (0.5 rpo) to x14.5 (4 rpo). This wide range of performance was due primarily to a small number of poor performers. A repeated measures ANOVA was conducted with Greenhouse-Geisser correction; there were two levels of the between-subjects factor group (practiced, naïve) and five levels of the factor modulation rate (0.25, 0.5, 1, 2 & 4). The 8-rpo data were excluded due to uniform ceiling performance. This analysis revealed a significant effect of rate (F_2.5,45.8_ = 32.50, p < 0.001), but no effect of group (F_1,18_ = 1.64, p = 0.217) and no rate-by-group interaction (F_2.5,45.8_ = 0.11, p = 0.936). This result indicates that most listeners are near asymptotic performance with relatively little practice.

Four out of 15 naïve listeners had at least one threshold that was a factor of two or more above the mean for practiced listeners. This poor performance in a subset of naïve listeners appeared to be more likely at high modulation rates (2 & 4 rpo) than low modulation rates (0.25 & 0.5 rpo), although one listener’s thresholds were elevated at all rates. This trend is opposite to that observed by Sabin et al. [27], who found greater evidence of improved spectral modulation detection at low than high modulation rates following practice. The observation of larger practice effects at high rates in the present paradigm should be treated cautiously, given the small number of listeners. However, if these findings are confirmed they would indicate that low-rate spectral modulation phase reversal thresholds could be a better clinical indicator of hearing deficit than high-rate spectral modulation phase reversal thresholds.

## General discussion

The purpose of the present report was to evaluate a measure of spectral modulation sensitivity that blends the traditional approaches of *spectral modulation detection threshold in dB* and *spectral modulation phase reversal threshold in rpo*. The first experiment provided support for estimating modulation phase reversal thresholds by adaptively varying spectral modulation depth in units of log(dB). Experiment 2 compared thresholds for spectral modulation detection and modulation phase reversal in dB. Results replicated the continued good spectral modulation detection thresholds at and above 8 rpo, as observed by Anderson et al. [14]. This result has been hypothesized to reflect temporal cues that support the detection of high-rate spectral modulation in the context of a spectrally flat standard. In contrast to spectral modulation detection, modulation phase reversal thresholds were at ceiling (60 dB) for 8 rpo, consistent with the idea that listeners were not able to use temporal cues under these conditions. While spectral modulation detection at 4 rpo and above may benefit from a temporal cue, performance for lower rates is no better than predicted based on spectral modulation phase reversal thresholds.

Experiments 2 and 3 evaluated sensitivity to spectral modulation as a function of frequency region and the presence of masking noise in flanking frequency regions. Off-frequency masking was negligible when spectral modulation was carried by two contiguous 1-oct bands, but maskers elevated thresholds by as much as a factor of 2.4 for one-band targets. Both datasets are consistent with less weight being given to bands at the spectral edges of the stimulus (0.125 & 8 kHz) than to bands in the middle region. While spectral weights were relatively flat for bands between 0.25 and 4 kHz when the target modulation was 0.25 and 0.5 rpo, increasing modulation rates were associated with increasing evidence of higher weights in the region of 2 kHz. This outcome is consistent with the frequency effects observed by Supin et al. [17] for modulation phase reversal thresholds in dB, but inconsistent with the modulation detection data of Eddins and Bero [19]. This discrepancy in results across paradigms raises the possibility that temporal cues available in the modulation detection paradigm obscure effects of carrier frequency, which are evident in the modulation phase reversal paradigm. Greater sensitivity to high-rate spectral modulation at mid and high carrier frequencies than at low carrier frequencies (< 2 kHz) could reflect frequency-dependent peripheral frequency resolution [28].

In the final experiment, spectral modulation phase reversal thresholds in dB were compared for highly practiced and naïve listeners. Thresholds for four out of the 15 naïve listeners were elevated relative to the practiced listeners for at least one modulation rate in the range of 2 to 4 rpo, with some indication of more consistent performance at lower rates. This range of variability for high-rate spectral modulation phase reversal thresholds within a population of young, normal-hearing adults suggests that clinical implementation of this task may be limited to a lower modulation rate (e.g., 0.5 rpo). Such a restriction would not necessarily be onerous since speech perception is thought to rely predominantly on relatively low-rate spectral modulations [2, 6, 7], and therefore the clinical relevance of results in this region may be particularly informative. In contrast to results obtained with high-rate spectral modulation, where spectral resolution is more likely to play a role in performance of hearing-impaired listeners [19], data on sensitivity to low-rate spectral modulation is thought to reflect a listener’s ability to combine spectral information across frequency.

## Supporting information

S1 Table Spectral modulation data 8_11_17.xlsx. Listener thresholds by experiment.
(XLSX)
